# Activity-Dependent Adenosine Release May Be Linked to Activation of Na^+^-K^+^ ATPase: An *In Vitro* Rat Study

**DOI:** 10.1371/journal.pone.0087481

**Published:** 2014-01-29

**Authors:** Robert Edward Sims, Nicholas Dale

**Affiliations:** School of Life Sciences, University of Warwick, Coventry, United Kingdom; University of Louisville, United States of America

## Abstract

In the brain, extracellular adenosine increases as a result of neuronal activity. The mechanisms by which this occurs are only incompletely understood. Here we investigate the hypothesis that the Na^+^ influxes associated with neuronal signalling activate the Na^+^-K^+^ ATPase which, by consuming ATP, generates intracellular adenosine that is then released via transporters. By measuring adenosine release directly with microelectrode biosensors, we have demonstrated that AMPA-receptor evoked adenosine release in basal forebrain and cortex depends on extracellular Na^+^. We have simultaneously imaged intracellular Na^+^ and measured adenosine release. The accumulation of intracellular Na^+^ during AMPA receptor activation preceded adenosine release by some 90 s. By removing extracellular Ca^2+^, and thus preventing indiscriminate neuronal activation, we used ouabain to test the role of the Na^+^-K^+^ ATPase in the release of adenosine. Under conditions which caused a Na^+^ influx, brief applications of ouabain increased the accumulation of intracellular Na^+^ but conversely rapidly reduced extracellular adenosine levels. In addition, ouabain greatly reduced the amount of adenosine released during application of AMPA. Our data therefore suggest that activity of the Na^+^-K^+^ ATPase is directly linked to the efflux of adenosine and could provide a universal mechanism that couples adenosine release to neuronal activity. The Na^+^-K^+^ ATPase-dependent adenosine efflux is likely to provide adenosine-mediated activity-dependent negative feedback that will be important in many diverse functional contexts including the regulation of sleep.

## Introduction

Adenosine is perhaps the most pervasive modulator in the brain, where it can act at a number of G-protein coupled receptors [1] to modulate neuronal and network activity [2]–[5]. For example adenosine is an endogenous somnogen and is very important for the homeostatic control of sleep [6]. Acting via A1 receptors adenosine universally mediates presynaptic inhibition of glutamatergic synapses [3]. It is increasingly apparent that the extracellular concentration of adenosine can be increased as a result of neural activity, allowing adenosine to mediate state-dependent actions that depend on prior activity in the nervous system [7]–[13]. Some of this adenosine arises from prior release of ATP from astrocytes. However there is evidence for direct adenosine release from neurons. In the cerebellum this arises from exocytosis, but in other brain regions, such as hippocampus and cortex, direct activity-dependent release of adenosine appears to be mediated via facilitative transporters [12].

The link between neural activity and the production of intracellular adenosine which can be transported into the extracellular space remains unclear. There has been a general idea that the metabolic load of neuronal signalling causes consumption of ATP with consequent production of intracellular adenosine; this would then be extruded from the cell by adenosine clearance mechanisms such as facilitative transporters. Together, these two systems would represent activity-dependent release of adenosine into the extracellular environment. Most of the resting metabolic load of the brain is consumed by the pumps that restore the differential concentration of Na^+^ across membranes [14]. An attractive hypothesis is therefore that activation of the Na^+^-K^+^ ATPase can cause rapid transporter-mediated release of adenosine. As this hypothesis has not been directly tested, we have used a combination of adenosine biosensing and Na^+^ imaging to directly evaluate the role of the Na^+^-K^+^ ATPase in activity dependent adenosine release. We have examined adenosine release mechanisms in primary motor cortex and the basal forebrain (BFB), a region connected to the control of slow wave sleep. In both areas we find that activation of the Na^+^-K^+^ ATPase is linked to the accumulation of extracellular adenosine.

## Methods

### Slice Preparation

300 µm-thick (400 µm-thick for imaging) coronal slices including the basal forebrain were obtained from 18–30-day-old, male, Sprague-Dawley rats. All animal handling was carried out in strict accordance with the UK Animals (Scientific Procedures) Act 1986 (licence PPL 80/2493) with all efforts made to minimise suffering. Animals were sacrificed by cervical dislocation and the brain was rapidly extracted and placed in a sub −4°C artificial cerebrospinal fluid (aCSF; see below for composition) containing an additional 10 mM MgCl_2_. Slices were cut on a Microm HM 650 V microslicer (Carl Zeiss, Welwyn Garden City, UK) and then transferred to a holding chamber at room temperature in standard aCSF composed of (in mM): NaCl, 124; KCl, 3; CaCl_2_, 2; NaHCO_3_, 26, NaH_2_PO_4_, 1.2; MgSO_4_, 1; glucose, 10; equilibrated with 95%∶5% O_2_∶CO_2_ to pH 7.4. Slices were incubated for at least one hour prior to initial experiments.

### Biosensor recording and analysis

Individual slices were placed on a nylon net, submerged in a recording chamber perfused with 32–33°C aCSF at a flow rate of 5–6 ml/min which was recycled, allowing sufficient run-out to waste during solution changes for different drug applications to avoid contamination of solutions. Microelectrode biosensors (Sarissa Biomedical, Coventry, UK) were carefully placed in the slice in pairs, one adenosine (ADO) sensitive and the other Null (lacking any enzymes), in BFB and cortex so that the active region was fully in the slice. We have previously published detailed accounts of the biosensor characteristics and use [15]–[19]. The Null sensors act as a control for any non-specific signals; the traces illustrated are the difference between the ADO and Null sensors. The ADO sensors will respond to adenosine, inosine and hypoxanthine and thus give a total purine signal, although most of this signal arises from adenosine in this application [19]. The biosensor signals were normalized to the sensitivity of the biosensor to 10 µM adenosine.

**Figure 1:**
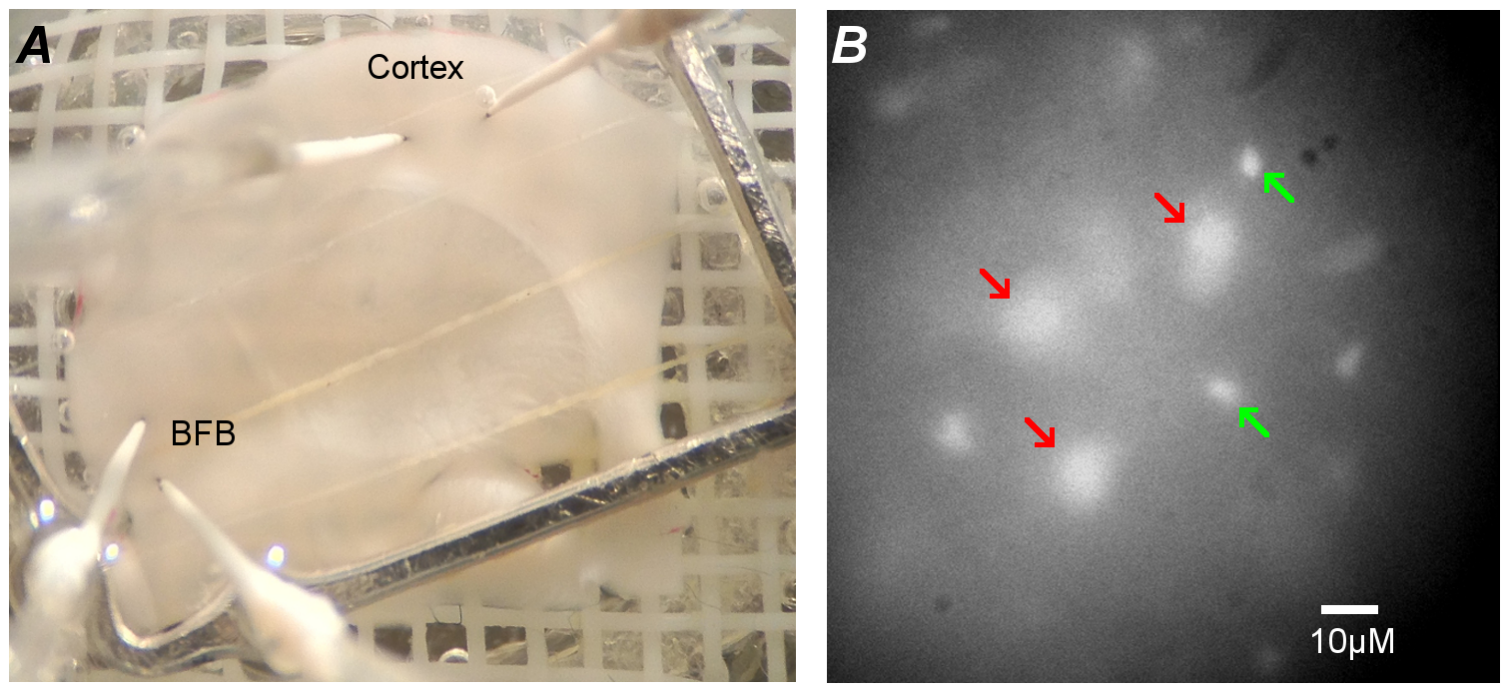
Biosensor placement and example of SBFI loading. A) Photograph of the slice in the holding chamber demonstrating biosensor placement in basal forebrain (BFB) and cortex. B) Representative example of SBFI loading at 340 nm excitation, showing loaded neurons (red arrows) and astrocytes (green arrows).

The areas of the BFB used were the horizontal arm of the diagonal band of Broca or substantia innominata. In the coronal slices at the same antero-posterior level as the BFB the cortex is primary motor cortex. The biosensors were placed into the middle of the cortical layer giving an approximate localization in layer 5 (Fig 1A). Initial insertion of biosensors resulted in a transient purinergic response (20–40 minutes), which was allowed to decay before experiments commenced. After the experiments were completed, the biosensors were withdrawn from the slice and calibrated with aCSF containing 10 µM adenosine, followed by aCSF with 10 µM serotonin to check that the sensor was adequately shielded and thus insensitive to other electro-active biological substrates. All drugs were bath applied by addition to the aCSF, and were tested to ensure they did not directly interfere with biosensor sensitivity.

Biosensor signals were measured on a Duo-Stat ME200+ potentiostat (Sarissa Biomedical, Coventry, UK) and acquired on a DT3010 data acquisition board (Data Translation, Bietigheim-Bissingen, Germany). Offline data analysis was done with customised software. Adenosine concentrations were measured at peak, and data are presented as mean ± standard error of the mean. We used a time of 10% peak to represent the start of concentration rises in order to discriminate from potential noise in the baseline. All statistics were carried out using unpaired students T-test.

### Measurement of intracellular Na^+^

The ratiometric dye, SBFI (sodium-binding benzofuran isophthalate; Invitrogen), was used to measure Na^+^. SBFI-AM was loaded into neurons and astrocytes via injection from a pipette of the AM dye into the centre of the slice (Fig 1B). After some 20 minutes neurons and astrocytes had taken up sufficient dye to allow measurements to commence. The slices were mounted in a flow chamber and visualized with an Olympus BX51 microscope through a 60× water immersion objective. An Andor Ixon EM-CCD was used to collect the images. Illumination at 340 and 380 nm was provided by a mercury arc lamp (Cairn Research) and a monochromator (Optoscan, Cairn Research). Metafluor software (Cairn Research) was used to control the experiments and store and analyze the data. Biosensors were placed into the slice, and the imaging regions arranged such that Na^+^ was measured close to the location of the biosensors.

### Zero Ca^2+^ aCSF

To remove Ca^2+^ signalling we modified standard aCSF by including 1 mM EGTA (ethylene glycol-bis(2-aminoethylether)-N,N,N′,N′-tetraacetic acid) and substituting Ca^2+^ with additional Mg^2+^. In the initial experiments with ouabain we also included cyclopiazonic acid at 20 µM, to block the Ca^2+^-ATPase in the endoplasmic reticulum and hence deplete intracellular stores. However we found that zero extracellular Ca^2+^ alone was sufficient to block the indirect actions of ouabain and did not include cyclopiazonic acid in later experiments.

## Results

**Figure 2:**
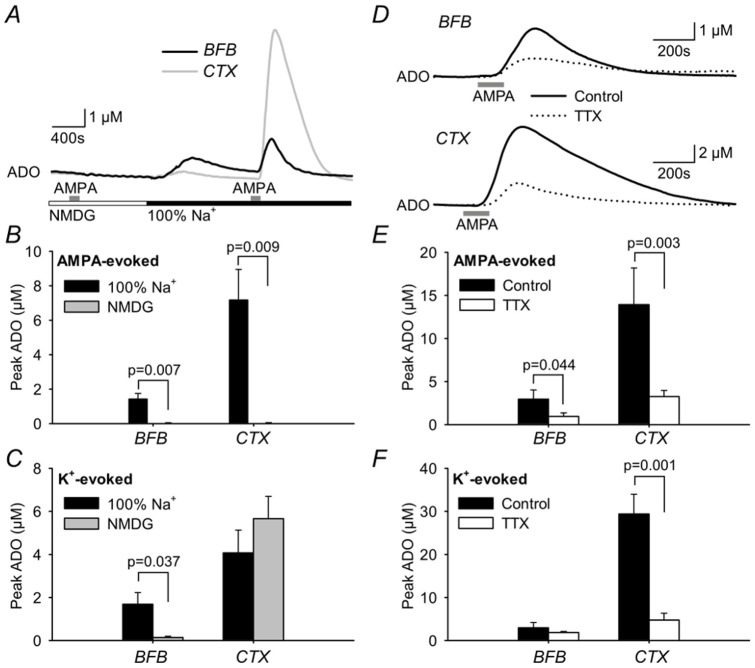
AMPA receptor evoked adenosine release depends upon a Na^+^ influx in cortex and basal forebrain. A) Adenosine biosensor recording, showing that when 80% of Na^+^ was substituted with NMDG^+^, AMPA (5 µM) could not evoke adenosine release in basal forebrain (BFB) or cortex (CTX). Re-introduction of Na^+^ restored AMPA-evoked adenosine release. B) Summary data showing that AMPA-evoked adenosine release in BFB and cortex is completely dependent on extracellular Na^+^ (n = 6). C) High K^+^-evoked adenosine release depends upon extracellular Na^+^ in BFB but not in cortex (n = 6). D,E) AMPA-evoked adenosine release was substantially blocked by TTX. f) TTX also blocked high K^+^-evoked adenosine release in cortex (control, n = 10; TTX n = 12). Statistical comparisons via Student's unpaired t-test.

We have previously shown that certain depolarizing stimuli, most notably activation of AMPA (2-amino-3-(3-hydroxy-5-methyl-isoxazol-4-yl)propanoic acid) receptors, reliably evoke adenosine release in the basal forebrain (BFB) and cortex [19]. As this release of adenosine is independent of both intracellular and extracellular Ca^2+^ [19], we tested whether an influx of Na^+^ might trigger adenosine release. Substitution of 80% of extracellular Na^+^ with NMDG, completely and reversibly blocked AMPA-evoked adenosine release in both the BFB and cortex (Fig 2A,B). The substitution of Na^+^ also blocked the ability of high K^+^ to evoke adenosine release in BFB but not in cortex (Fig 2C). The difference in Na^+^-dependence of high K^+^-evoked AMPA release between BFB and cortex is curious, and may be related to the different types of cells and relative proportions of astrocytes and neurons in the two areas.

**Figure 3:**
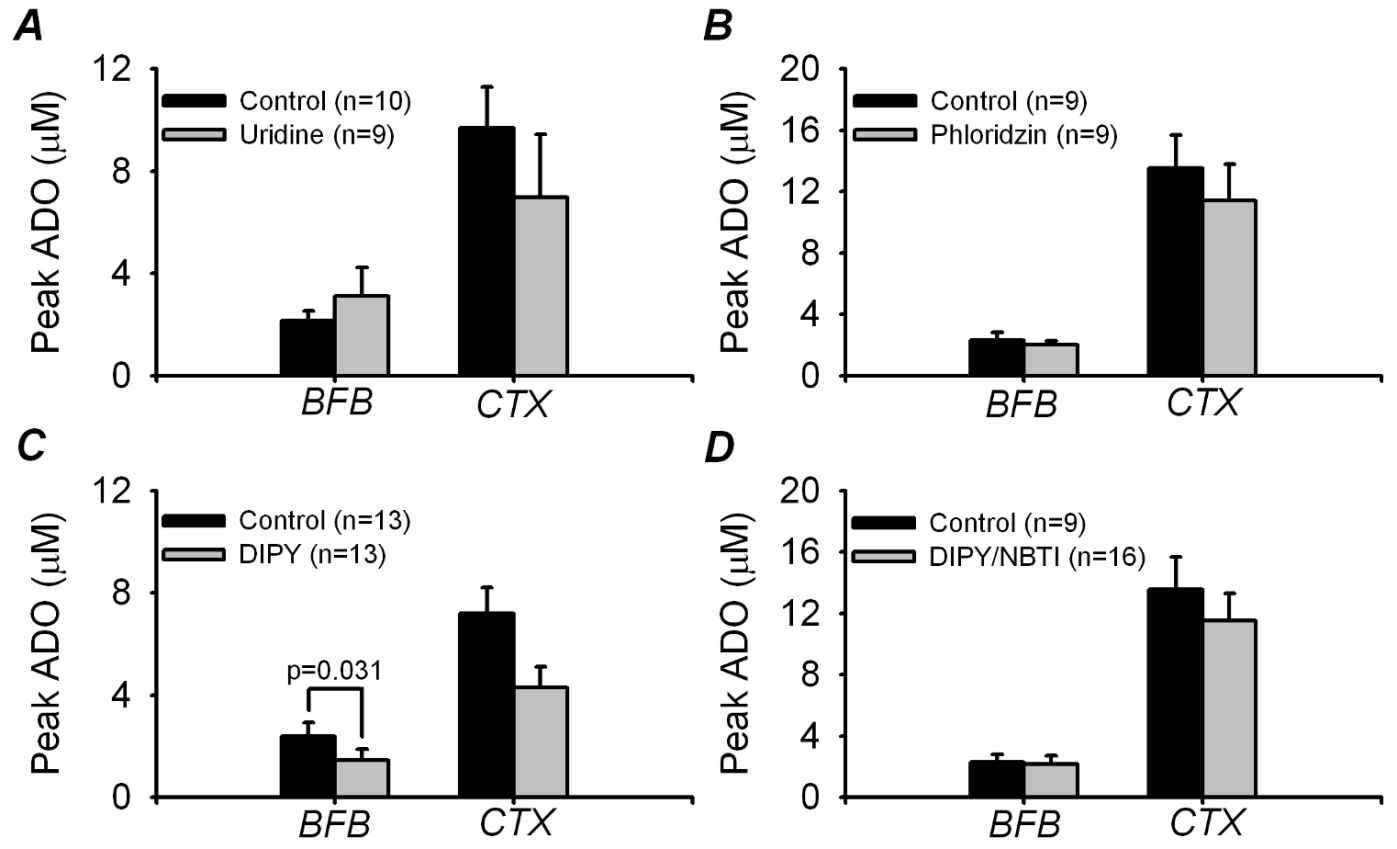
Na^+^-dependent concentrative nucleoside transporters (CNTs) and equilibrative nucleoside transporters (ENTs) do not play a role in AMPA-evoked adenosine efflux. A) Preincubation of slices (for 1 h) with a competing substrate for the CNTs, 100 µM uridine, had no effect on adenosine release in basal forebrain (BFB) or cortex (CTX). B) Application of 200 µM phloridzin, a general blocker of Na^+^-dependent transporters including the CNTs had no effect on adenosine release either. C) Addition of the ENT blocker dipyramidole (DIPY; 20 µM) prior to AMPA application caused a significant reduction in BFB adenosine release but not CTX. D) Application of both 20 µM DIPY and 10 µM NBTI (another ENT blocker) however did not significantly reduce adenosine release in BFB or CTX.

The AMPA-evoked adenosine release was also sensitive to TTX, which substantially, but not completely (>60% block), inhibited adenosine release in both BFB and cortex evoked by AMPA and high K^+^ (Fig 2D–F). The Na^+^-dependent adenosine efflux could occur via the Na^+^ linked concentrative nucleoside transporters (CNTs). Although the CNTs normally take up adenosine from the extracellular space (because of the Na^+^ gradient from outside to inside the cell), the accumulation of intracellular Na^+^ and depolarization from AMPA receptor activation could transiently reverse the driving force on the CNTs in dendritic spines and cause an efflux of adenosine as can happen for glutamate under some circumstances [21] However neither preincubation with 100 µM uridine for 1 hour (a competitive substrate for the CNTs [20]; Fig 3A) nor 200 µM phloridzin (an inhibitor of many Na^+^-dependent transport processes [22]; Fig 3B) affected the AMPA-evoked adenosine efflux, leading us to discount this possibility.

An alternative pathway for release of adenosine could be via the equilibrative nucleoside transporters (ENTs). We therefore applied 20 µM dipyridamole (DIPY; Fig 3C) alone or 20 µM DIPY plus 10 µM NBTI (nitrobenzylthioinosine; blockers of the ENTs; Fig 3D). DIPY alone reduced adenosine release in response to AMPA application from 2.4±0.5 and 7.2±1.0 µM (n = 13, BFB and cortex respectively) in the control to 1.5±0.4 µM and 4.3±0.8 µM (n = 13, BFB and cortex respectively). In the BFB, but not the cortex, this reduction was statistically significant: p = 0.031, DIPY vs control for BFB; and p = 0.104, DIPY vs control for cortex (Student's t-test). However the combination of NBTI and DIPY was without any significant effect in either BFB or cortex: control, 2.3±0.5 and 13.5±2.1 µM (n = 9, BFB and cortex respectively); NBTI/DIPY, 2.2±0.5 (n = 16) and 11.5±1.72 µM (n = 17), BFB and cortex respectively. We think that the statistical significance of the effect of DIPY on its own in BFB is most probably a type 1 statistical error (false rejection of the null hypothesis) as we could not repeat this result with the combination of transport blockers that should have been more effective at reducing adenosine efflux (e.g. [13], [17]). It therefore seems safest to conclude that the adenosine efflux evoked by AMPA application in BFB and cortex is not mediated via transporters that are sensitive to NBTI/DIPY.

**Figure 4:**
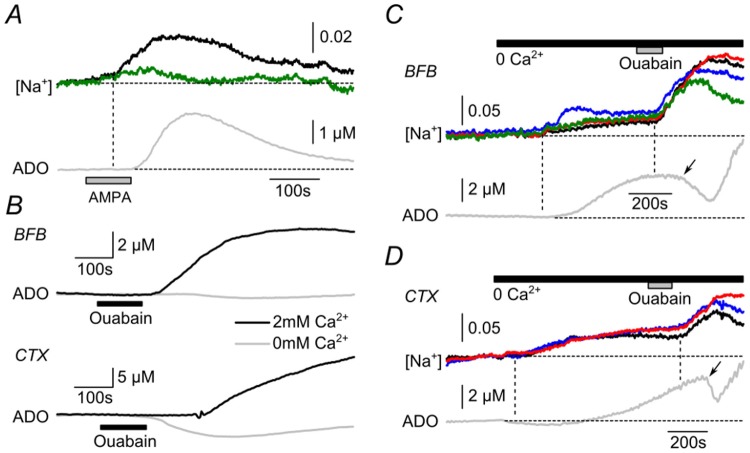
Adenosine release depends upon activation of the Na^+^-K^+^ ATPase via accumulation of intracellular Na^+^. A) Simultaneous measurement of intracellular concentration of Na^+^ with SBFI, and adenosine release during AMPA receptor activation (5 µM AMPA) in basal forebrain. Note the increase in intracellular Na^+^ precedes the adenosine release recorded by the biosensor (ADO). Black trace, neuron; green trace, astrocyte. B) Application of 100 µM ouabain caused adenosine release in BFB and cortex in presence of normal extracellular Ca^2+^. In the absence of extracellular Ca^2+^ ouabain caused either no change in extracellular adenosine or a reduction in adenosine. C) In BFB, application of zero Ca^2+^ aCSF caused an increase in intracellular Na^+^ in neurons (back, blue, red) and astrocytes (green) traces as measured by SBFI. Simultaneous recording with an adenosine biosensor showed an increase in extracellular adenosine following shortly after this increase in intracellular Na^+^. When ouabain was applied, intracellular Na^+^ levels increased in all cells, but the extracellular adenosine levels initially fell (arrow), before increasing as ouabain washed out. D) In cortex a similar pattern was seen: zero Ca^2+^ aCSF caused an increase in intracellular Na^+^ in neurons (back, blue, red) accompanied after a short delay by the release of adenosine measured by the nearby biosensor. When 100 µM ouabain was applied, adenosine levels fell (arrow) despite the increase in intracellular Na^+^.

We next used the ratiometric dye SBFI, to measure the accumulation of intracellular Na^+^ in both astrocytes and neurons, identified on the basis of their morphology and size (Fig 1B). AMPA application readily caused a detectable increase of intracellular Na^+^ (peak change in fluorescence ratio 0.034±0.008 in neurons and 0.026±0.005 in astrocytes; Fig 4A). This increase commenced after 100±14 s, n = 8 (time to 10% of peak rise) after the application of AMPA and was followed after a delay of about 90 s by a rapid increase in extracellular adenosine (achieved 189±13 s, n = 8, time to 10% of peak rise, after the application of AMPA). Both the adenosine biosensor recordings and Na^+^ imaging can resolve events on a second to second timescale e.g. [8], [11], [23]. This substantial delay between the increase in Na^+^ and the release of adenosine is therefore biological in origin and suggests that the link between these two events is indirect. This is consistent with our conclusion that a direct Na^+^-mediated transport mechanism is not involved in the Na^+^-dependent adenosine release.

The Na^+^ influx will activate Na^+^-K^+^ ATPases and cause additional consumption of ATP. The enzyme *adenylate kinase* will utilize ADP to convert it back to ATP with the production of AMP. This AMP can then be broken down to adenosine by the 5′ nucleotidase. These coupled reactions are required to keep intracellular ADP levels low and thus keep the ATP∶ADP ratio very high, thereby allowing ATP hydrolysis to drive coupled reactions in the cell. ATP hydrolysis thus results in increases in the concentration of intracellular adenosine. In neurons, which have very little *adenosine kinase* [24], which converts adenosine to AMP, this adenosine could reach sufficient concentrations to then enter the extracellular space via an, as yet uncharacterized, transporter. It is therefore possible to make a plausible and clear prediction that activation of the Na^+^-K^+^ ATPases may cause an efflux of adenosine.

The obvious tool to test involvement of the Na^+^-K^+^ ATPase is ouabain. However, by reducing the activity of the Na^+^-K^+^ ATPases, ouabain causes general depolarization of neurons and release of many transmitters with consequent multiple indirect actions. As we have described in hippocampus [17], brief application of ouabain (100 µM) under control conditions increased intracellular Na^+^ and caused profound adenosine release (4.3±1.0 µM, n = 3 in BFB; and 38.6±4.9 µM, n = 3 in cortex, Fig 4B). However part of this adenosine release may have arisen from the indiscriminate activation of neurons and networks caused by the powerful depolarising actions of ouabain.

We therefore exploited the Ca^2+^-independence of adenosine release [19] in our experiments as a way of preventing these indirect actions of ouabain. When extracellular Ca^2+^ was removed from the aCSF, ouabain did not cause adenosine release, confirming that the previously observed release (in the presence of extracellular Ca^2+^) was an indirect consequence of the non-specific neuronal and network activation caused by ouabain (Fig 4B). Indeed, ouabain application in the absence of extracellular Ca^2+^ often caused a decrease in extracellular adenosine (Fig 4B, see below). This observation meant that we could use ouabain as a tool to probe the cell-specific role of the Na^+^-K^+^ ATPase under conditions of zero extracellular Ca^2+^.

We reasoned that following an increase in intracellular Na^+^ and consequent adenosine release, application of ouabain, by reducing ATP consumption via the Na^+^-K^+^ ATPases should inhibit the adenosine efflux. Exposure of slices to zero Ca^2+^ aCSF caused a gradual increase in intracellular Na^+^ in the BFB and cortex as measured by SBFI fluorescence (Fig 4C,D). This is expected given that hypocalcaemic conditions have long been known to increase membrane excitability [25] and increase Na^+^ influx through channels and exchangers [26], [27], and removal of extracellular Ca^2+^ will open pannexin and connexin hemichannels. These hemichannels are non-selective large conductances present in a variety of neurons [28]–[31] and would allow Na^+^-entry.

This modest increase in intracellular Na^+^, which we observed on removal of extracellular Ca^2+^, was accompanied after a short delay by an increase in extracellular adenosine (mean increase in BFB and cortex respectively 1.0±0.4 µM, n = 12 and 7.1±1.6 µM, n = 12). Brief application of 100 µM ouabain resulted in a further rapid increase in intracellular Na^+^ indicating that ouabain had blocked the Na^+^-K^+^ ATPase (Fig 4C,D). Crucially this increase in intracellular Na^+^ was followed by a rapid drop in extracellular adenosine (Fig 4C,D, arrow, mean decrease adenosine concentration in BFB and cortex respectively 0.4±0.1 µM, n = 11 and 5.8±1.4 µM, n = 12). As the actions of ouabain washed out, intracellular levels of Na^+^ started to fall (Fig 4C,D), and the extracellular concentration of adenosine rose as Na^+^-K^+^ ATPase activity resumed and possibly other Na^+^-dependent ATP-consuming processes became activated.

**Figure 5:**
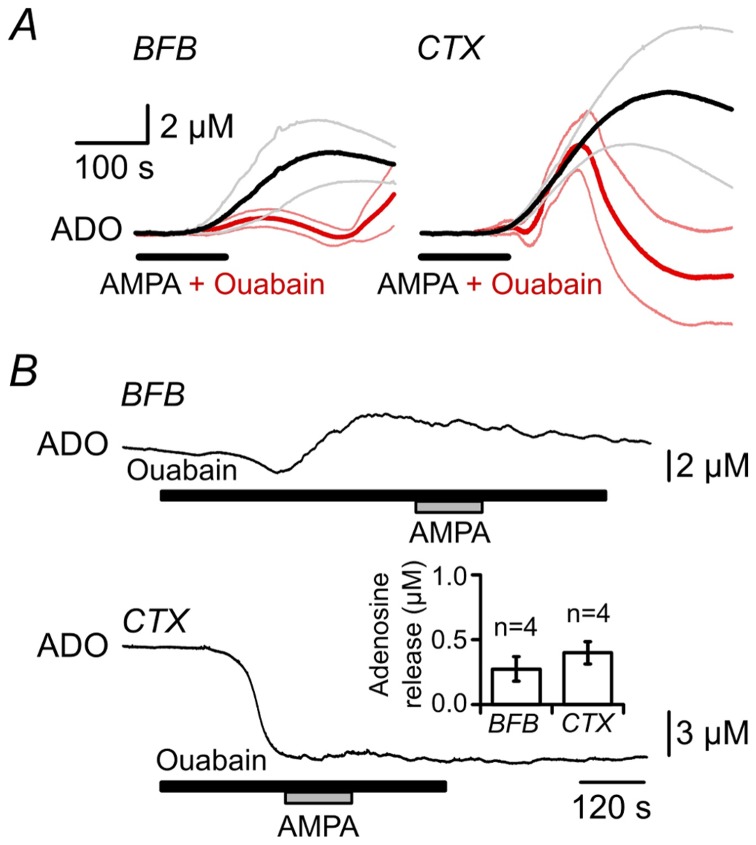
Inhibition of the Na^+^-K^+^ ATPase by ouabain reduces AMPA-evoked adenosine release. Adenosine biosensor recordings from BFB and CTX from slices were perfused with zero Ca^2+^ aCSF. A) Comparison of 5 µM AMPA application (black lines) alone with simultaneous application of 100 µM ouabain and 5 µM AMPA (red lines). The data are presented as averages ±SD (thin lines) of 4 experiments for each condition. When applied simultaneously with AMPA the ouabain caused the adenosine release to be cut short and truncated compared to the adenosine release evoked by AMPA alone. B) Preincubation with 100 µM ouabain, once the adenosine levels had stabilized, greatly reduced the response to AMPA (5 µM). Inset: Summary graph showing that prior treatment with ouabain greatly inhibits the adenosine release evoked by 5 µM AMPA (mean ± sem).

We next tested whether ouabain altered the adenosine efflux stimulated by activation of AMPA receptors. Once again, to reduce the secondary effects of ouabain, we performed these experiments under conditions of zero extracellular Ca^2+^. We used two experimental paradigms. Firstly a 2 minute simultaneous application of AMPA plus 100 µM ouabain was compared to 2 minute application of AMPA alone. We found that simultaneous application of ouabain greatly reduced the amplitude of the AMPA-evoked adenosine release, and cut short the duration of adenosine release in both cortex and BFB compared to AMPA alone (Fig 5A). In the BFB, the adenosine levels increased markedly as the ouabain washed out and the Na^+^-K^+^ ATPase became active (compare to Fig 4C). In cortex the adenosine levels went below the preceding baseline, probably because the conditions of zero Ca^2+^ cause an increase in intracellular Na^+^, increased activation of the Na^+^-K^+^ ATPase and consequent increase in the adenosine tone (compare to Fig 4D), which was then additionally counteracted by ouabain. Secondly, we applied 100 µM ouabain prior to the application of AMPA. Although ouabain altered the extracellular adenosine levels, once these had stabilized, subsequent application of AMPA caused very little further adenosine release (Fig 5B, compare inset to Fig 2). Overall these two experimental paradigms demonstrated that the efflux of adenosine resulting from the activation of AMPA receptors was greatly reduced by ouabain.

## Discussion

Infusion of adenosine into a single hippocampal pyramidal cell will cause depression of the synaptic inputs to that cell via activation of A1 receptors, suggesting that elevated levels of intracellular adenosine will readily be transported into the extracellular space [32], [33]. On this basis, any process that increases endogenous adenosine levels in neurons should therefore also affect its extracellular concentration. Metabolic processes that consume ATP will cause an increase in intracellular adenosine. As a very large proportion of the brain's energy budget is consumed by neurons pumping out the Na^+^ that has entered during signalling [14], the activation of the Na^+^-K^+^ ATPases is an attractive candidate mechanism to underlie a significant degree of activity-dependent adenosine efflux. The adenosine concentrations observed in this study are slightly higher than the reported EC50s of the adenosine A1 receptor of around 1 µM, measured with respect to presynaptic inhibition of neurotransmission ([15], [34], [35]. These levels are also higher than the levels of adenosine release seen in response to neuronal activation by stimulation of axon tracts in cerebellum [9], [11], [13] and hippocampus [13]. Nevertheless these are not saturating concentrations with respect to the A1 receptor, and the larger amount of release is most probably due to the fact that AMPA application in the bathing medium will cause a global excitation that likely exceeds what will be observed *in vivo*.

### Colocalization of AMPA receptors and the Na^+^-K^+^ ATPase

Our study suggests that activation of AMPA receptors will give a discrete and reproducible adenosine efflux that depends on the additional activation of the Na^+^-K^+^ ATPase resulting from the Na^+^ influx through the AMPA channel. This link between activation of AMPA receptors and adenosine release may be explained by the subcellular localization of the Na^+^-K^+^ ATPase and its physical proximity to AMPA receptors. These pumps are located in dendritic spines [36] and colocalize with AMPA receptors [37]. There are also reports that AMPA receptor subunits coimmunoprecipitate with subunits of the Na^+^-K^+^ ATPase suggesting that they form a complex [38]. The close proximity between these two molecules may plausibly help to ensure that the Na^+^ influx resulting from AMPA receptor activation will efficiently activate the Na^+^-K^+^ ATPase with a resulting efflux of adenosine. We propose that adenosine efflux linked to the activation of the Na^+^-K^+^ ATPase during neural signalling may be important in many contexts: the setting of general excitability levels in the brain; responses to seizure activity; and the release of adenosine connected to the control of sleep.

### Network or cellular effects?

AMPA will excite many cells which could release further transmitters that might contribute to adenosine release. This is unlikely to significantly affect our results as the AMPA-evoked adenosine release persists in the absence of extracellular Ca^2+^ which would block transmitter release from neurons and hence prevent these indirect effects. Similarly, whilst 20% normal extracellular Na^+^ after substitution with NMDG could be expected to reduce depolarisation of cells by AMPA and reduced network activity, depolarisation with high K^+^ should be expected to cause widespread depolarisation. Finally, the fact that ouabain, also a potent depolarising influence, reduces adenosine release in the absence of extracellular Ca^2+^ means a network effect is improbable. A potential confounding factor is that ouabain will cause widespread collapse of Na^+^ and other ionic gradients across the cells. However, neuronal depolarisation, swelling and alteration of ion concentrations are likely to be modest in the short timescales within which we observe concurrent Na^+^ influx and adenosine release. For example Figures 4C and 4D show that intracellular Na^+^ levels have not yet achieved a plateau at the time we observed the fall in extracellular adenosine. After a protracted period of exposure of around 5–10 minutes, ouabain can lead to a spreading depression-like event, which presages irreversible cell damage [39], [40].

### Other mechanisms of activity-dependent adenosine release

Activation of the Na^+^-K^+^ ATPase may explain why spiking activity of neurons has been linked to adenosine release [12]. Nevertheless this mechanism is unlikely to be the only mechanism for activity dependent direct adenosine release from neurons for three reasons. Firstly, adenosine appears to be released via exocytosis in the cerebellum [8], [9], [11]. Secondly, in hippocampus, AMPA-evoked adenosine release exhibits Ca^2+^ dependence [13], unlike that seen in the BFB and cortex [19]. Thirdly, although the adenosine efflux is discrete and reproducible, there is a significant delay between the accumulation of intracellular Na^+^ (which commences as soon as the AMPA receptor channel is opened) and the efflux of adenosine (∼90 s in our experiments). This delay presumably reflects the dynamics of the intracellular metabolism that produces adenosine on hydrolysis of ATP. Examples of activity-dependent adenosine release show that it is produced with only a very short latency following neuronal activation in cerebellum [8], [9], [11] and hippocampus [41]. This disparity in timing suggests that there must be other mechanisms that more rapidly induce activity-dependent adenosine efflux. The Na^+^-dependent mechanism that we describe in this paper may be more suited to slower activity dependent feedback that results from integrating all sources of excitatory activity in a cell to provide quasi-autocrine regulation of excitability.

Our study is the first experimental test of this hypothesis and documents that activation of AMPA receptors will give a discrete and reproducible adenosine efflux that depends on the additional activation of the Na^+^-K^+^ ATPase resulting from the Na^+^ influx through the AMPA channel. The Na^+^-K^+^ ATPase, regarded until now as primarily controlling the intracellular milieu, must additionally be considered as an important regulator of extracellular adenosine. As Na^+^-K^+^ ATPases are expressed in all cells of the brain this is likely to be a very general mechanism linking neural activation to adenosine release.

By contrast to neurons, astrocytes express high levels of adenosine kinase [24]. This enzyme by converting adenosine to AMP keeps the intracellular level of adenosine very low in astrocytes [42], [43] and may also help to buffer any increases in adenosine caused by activation of ATP consuming processes. The expression of adenosine kinase in astrocytes may limit the role of the Na^+^-K^+^ ATPase in causing adenosine release from these cells. Nevertheless the role of astrocytes in indirectly producing extracellular adenosine via ATP release remains important and we have recently shown that in hippocampus, transporter-mediated and astrocyte-mediated adenosine release are roughly equal in magnitude, but differ in their kinetics, the former being faster and briefer than the latter [13].
